# Hypogonadism, Type-2 Diabetes Mellitus, and Bone Health: A Narrative Review

**DOI:** 10.3389/fendo.2020.607240

**Published:** 2021-01-18

**Authors:** Vittoria Russo, Rui Chen, Reina Armamento-Villareal

**Affiliations:** ^1^ Division of Endocrinology, Diabetes, and Metabolism, Department of Medicine, Baylor College of Medicine, Houston, TX, United States; ^2^ Department of Medicine, Michael E. DeBakey VA Medical Center, Houston, TX, United States

**Keywords:** insulin resistance, osteoporosis, type 2 diabetes mellitus, hypothalamic-pituitary-gonadal axis, hypogonadotropic hypogonadism

## Abstract

One of the complications from chronic hyperglycemia and insulin resistance due to type 2 diabetes mellitus (T2DM) on the hypothalamic-pituitary-gonadal axis in men is the high prevalence of hypogonadotropic hypogonadism (HH). Both T2DM and hypogonadism are associated with impaired bone health and increased fracture risk but whether the combination results in even worse bone disease than either one alone is not well-studied. It is possible that having both conditions predisposes men to an even greater risk for fracture than either one alone. Given the common occurrence of HH or hypogonadism in general in T2DM, a significant number of men could be at risk. To date, there is very little information on the bone health men with both hypogonadism and T2DM. Insulin resistance, which is the primary defect in T2DM, is associated with low testosterone (T) levels in men and may play a role in the bidirectional relationship between these two conditions, which together may portend a worse outcome for bone. The present manuscript aims to review the available evidences on the effect of the combination of hypogonadism and T2DM on bone health and metabolic profile, highlights the possible metabolic role of the skeleton, and examines the pathways involved in the interplay between bone, insulin resistance, and gonadal steroids.

## Introduction

The frequent association between hypogonadism and metabolic disorders, such as obesity, insulin resistance, metabolic syndrome (MetS), and type 2 diabetes mellitus (T2DM), is well-known and has been described in multiple studies (1–7). In particular, hypogonadism is common in men with T2DM, and about one-third of men with T2DM men have low serum testosterone (T) levels (8). However, other estimates show that the prevalence of low T could be as high as 64% in men with T2DM (8, 9). The hypothetical mechanism for this association has been suggested in a study by Dhindsa et al. showing that as much as 75% of men with T2DM have either low or normal luteinizing hormone (LH) or follicle stimulating hormone (FSH) or both indicating suppression of the hypothalamic-pituitary-gonadal axis likely from chronic hyperglycemia (8). On the other hand, some studies also suggest that the association between T2DM and hypogonadism could be bidirectional. This is because low T is commonly found in men with T2DM, while men with T deficiency are at increased risk to develop impaired glucose tolerance (2, 10). It has been reported that T deficiency is predictive of an increased risk of developing incident T2DM (11–14), while men with high T levels have a 42% lower risk of developing T2DM (10). Regardless of the etiology of low T, the coexistence of hypogonadism and T2DM could be detrimental to bone as each is associated with increased fracture risk.

Low T levels is a risk factor for age-related decline in bone mass and increase in fragility fractures in men, making androgen deficiency an important risk factor for osteoporosis (15, 16). On the other hand, substantial epidemiological evidence also show that people with T2DM have an increased risk of fractures compared to those without diabetes despite a normal or high normal bone mineral density (BMD) (17–21). There are many factors that may contribute to this paradox, such as lower bone turnover, increased accumulation of advanced glycation end products (AGEs) in bone, increased fall risk from neuropathic and ophthalmologic complications of longstanding diabetes, and decreased physical activity (22, 23). Meanwhile, T exerts an anabolic effect on bone by its effect in stimulating osteoblasts differentiation and proliferation (24, 25). Thus, it is expected that the frequent presence of low T concentrations in the diabetic population also contributes to impaired bone strength in men with T2DM (26).

Considering the individual effect of T2DM and hypogonadism on bone health and their association with fragility fractures, it is possible that the combination of the two may have a worse effect on bone. Given the high and increasing prevalence of T2DM, and the high rate of concurrent hypogonadism in these patients, a significant number of men could be at a greater risk for fractures if the presence of both conditions confers an even higher risk than either one alone. While the metabolic effects of hypogonadism and T2DM in men have been well investigated, to date, there is little information on the bone health of these subjects and how these conditions may be interconnected. In this review, we examine the impact of the combination of hypogonadism and T2DM on bone health, and discuss the pathways involved in the interplay between insulin resistance and hyperglycemia, with gonadal hormones and bone metabolism.

## Methodology

A literature search of studies detailing results from cellular and animal experiments, clinical trials, meta-analysis, systematic and narrative reviews was performed in PubMed using the search terms as “T2DM,” “hypogonadism,” “testosterone,” “bone,” “insulin resistance,” “insulin sensitivity,” and “bone turnover markers.” Additional studies were identified from cross-references of the articles found. In total, 309 unique publications were found from 1969 to 2020 and 223 are included in this narrative literature review. We included all manuscripts investigating the relationship between hypogonadism and T2DM and their effect on bone, either separately or in combination. Also included in this report are publications discussing the role of insulin signaling in regulating bone metabolism and sex steroid production which we believed are relevant to this manuscript.

## Hypogonadotropic Hypogonadism and Type 2 Diabetes Mellitus

One-third to two-thirds of all men with T2DM have subnormal total or free T concentrations, and ~75% of them have low or inappropriately normal levels of LH and FSH suggesting hypogonadotropic hypogonadism (HH) (8, 13, 22). The common association between low T and T2DM has been confirmed in several studies (8, 27, 28). For instance, a cross-sectional study of 355 men aged > 30 years old with T2DM reported that 42% of these subjects had low T and another 25% had borderline low T levels (28). In a cross-sectional survey of 580 men with T2DM with a mean age of 65 (including patients with a range of age from < 40 years to > 80 years), 43% had reduced total T levels, while 57% had low calculated free T, which were both inversely related to age (27). Dhindsa et al. demonstrated that 33% of men with T2DM aged 28 – 80 yr, were hypogonadal, as shown by significantly lower levels of free T measured by equilibrium dialysis (8). Similarly, a high prevalence of HH was also found in younger men with T2DM between the ages of 18 and 35 years (14).

The possible pathophysiological mechanism underlying the cause of T2DM induced HH is not yet defined but is likely multifactorial. Some of the proposed mechanisms involve obesity, inflammation and insulin resistance (22). Obesity, which often accompanies T2DM, is also associated with low total and free T concentrations in men. In fact, in obese patients free T levels show an inverse correlation with BMI, while estradiol (E2) levels are increased in these subjects and positively correlated with BMI (29–31). The hypothalamic and pituitary mechanisms regulating LH and FSH release are further suppressed by the increased levels of E2 and leptin in obese patients and, as consequence, T concentrations are decreased in these subjects (29, 32).

Chronic inflammation can be considered as another mechanism that may promote the suppression of hypothalamic-pituitary-gonadal axis and HH in T2DM, as documented by studies in experimental animals and *in vitro* reporting that inflammatory mediators, such as tumor necrosis factor-α (TNF-α), and interleukin-1β (IL-1β), which are generally increased in T2DM, obesity, and MetS, are able to suppress hypothalamic gonadotrophin-releasing hormone and LH secretion (33, 34). Moreover, inflammation related mediators contribute to insulin resistance (35), which in turn plays a role in the development of HH in T2DM as shown by the decreased T concentration and LH and FSH levels resulting from the selective deletion of the insulin receptor from neurons in the central nervous system of mice (36).

The above data suggest that in subjects with HH and T2DM, the increased adiposity, together with a reduction in free T and a simultaneous reduction in muscle mass may contribute to develop a proinflammatory state and insulin resistance (22). However, there is also the possibility of a genetic predisposition as suggested by a recent study showing that rare isolated HH genes variants can frequently predispose to adult onset HH with acquired mild hormonal deficiencies (37).

Considering the frequent occurrence of HH in T2DM, although majority of the studies did not report LH and FSH levels in patients with hypogonadism and T2DM, we assume that a higher percentage of these subjects could have HH. The relationship between T and T2DM risk has been examined in meta-analysis including 43 prospective and cross-sectional studies comprising a total of 6,427 men. This study found that men with higher T levels had a 42% lower risk of T2DM compared with those with lower concentrations (10). In a cross-sectional study of 267 unselected men with T2DM, fasting blood glucose was inversely correlated with total T levels, and significantly higher in men with severe hypogonadism in comparison to patients with normal T (38). Other investigators suggest that the onset of T2DM may be preceded by hypotestosteronemia (12), making the androgen deficiency a possible risk factor for the development of T2DM (39).

## Hypogonadism and Bone

Hypogonadism is a well-established cause of osteoporosis in men (40). Decreased androgen levels have been linked to low BMD and an increase in fracture risk in men (15, 16, 22, 41). Hypogonadal men have reduced cortical and trabecular BMD compared to controls (15). Hypogonadism in adults leads to bone loss, while hypogonadism before puberty results in the failure to achieve peak bone mass (42, 43). T deficiency has been reported in over half of elderly men with a history of hip fracture (44, 45). Hypogonadism is associated with increased levels of bone turnover markers, mainly those of bone resorption (46, 47). In a study on 792 men, aged 50–85 years old, hypogonadal elderly subject had increased bone resorption not adequately matched by an increase in bone formation leading to bone loss (48).

T affects the male skeleton in several ways (47, 49–56). Firstly, androgens contributes to bone size by its effect on periosteal apposition, which explains the wider or bigger bones in men compared to women (57, 58), and to some extent to bone mass through increasing muscle mass (59). Having a bigger bone size denotes better bone geometry which translates to improved bone strength, due to better capacity to adapt to higher mechanical load and greater resistance to compression forces that triggers fractures (59, 60).

Secondly, T the major androgen, is the primary source of E2 in men. Through its conversion to E2 by the enzyme aromatase in adipose tissue, T appears to play an indirect but important role in mediating age related bone loss in men (61, 62). At the cellular level, estrogen is able to suppress bone resorption by decreasing osteoclasts formation and activity, and increasing osteoclasts apoptosis (63–65). E2 has long been considered a key regulator of bone metabolism not only in women, but also in adult male skeletal homeostasis (66–68). In a study on 93 healthy men over 55 years of age, BMD at various sites correlated with serum E2 levels, and inversely with serum T levels (69). Data from both observational and epidemiological studies in men have documented that BMD and bone turnover markers were more closely correlated with serum estrogen levels than serum T levels, and that the link between bone loss and fractures was stronger with E2 rather than T (62, 67, 70). Additionally, multivariate analyses showed that serum bioavailable estrogen level was a consistent independent predictor of BMD in men (62). Moreover the changes in BMD from androgen therapy correlate with the changes in E2 but not with the changes in T suggesting that the effect of androgen replacement on BMD is mediated by its conversion to estrogen (71). Studies that directly manipulated levels of T and E2 to assess their independent effects on the changes in BMD and markers of bone turnover, showed an increase in resorption markers and bone loss in the absence of both hormones; these events were however prevented by E2 supplementation but not by T (72, 73). Another study in which endogenous sex steroids were pharmacologically suppressed and followed by exogenous replacement of each hormone to tease out the effect of one over the other confirmed the dominant effect of estrogen, but not T, on bone resorption, with an estimate that that in men, estrogen accounts for over 70% of the total effect of sex steroids on bone resorption (66). Since T is the substrate from which majority of circulating E2 (over 80%) in men is derived (74), men with low T often have concurrently low E2 so the effects of low E2 on bone metabolism is indirectly from low T.

Thirdly, T has a separate and distinct anabolic effect because of its ability to stimulate osteoblastic differentiation and proliferation (25). In fact, both androgen and estrogen receptors are detected in several bone cells including osteoblasts, osteoclasts, and mesenchymal stromal cells, which differentiate into osteoblasts (73, 75). *In vitro* studies have shown that treatment with androgens result in osteoblasts differentiation, proliferation, along with bone matrix production and mineralization (76). T increases the lifespan of osteoblasts by decreasing apoptosis, mainly through its action on IL-6 production (77, 78), and is able to stimulate the proliferation of osteoblasts progenitors and the differentiation of mature osteoblasts (25, 78–80). However, how this anabolic effect of androgens on bone influences the skeleton separately from the antiresorptive effect of E2 is unclear. *In vitro*, both sex steroids have shown to exert antiapoptotic effects on osteoblasts and osteocytes, and the loss of estrogens or androgens shortens the lifespan of osteoblasts and osteocytes in gonadectomized mice from either sex (79). Also, because of the effect of androgens in stimulating osteoblasts proliferation and differentiation, increase in osteoprotegerin (OPG), a product of the osteoblasts, will follow resulting in decrease osteoclasts formation and bone resorption (81).

Finally, T could have important nonskeletal effects. Data from the Dubbo Osteoporosis Epidemiology study, showed that serum T levels predicted fracture risk in elderly men independent of BMD, suggesting an important role for T in modulating nonskeletal factors, such as muscle strength, which could be impaired in the presence of insufficient T levels predisposing to falls and fractures (82). Without question, the beneficial effects of T replacement therapy on bone mass in men with gonadal failure is well-established (47, 49–52). T replacement therapy is associated with a reduction in markers of bone turnover and a significant in increase of BMD in young and elderly men with hypogonadism (52–54). Increases of in vertebral and hip BMD after T treatment were seen in most studies (52, 55, 56). More importantly, improvement in bone quality has been reported in hypogonadal men on T replacement (56, 83).

While a symptom-specific threshold level for T has been identified for certain symptoms of hypogonadism such as: 1) loss of libido and vigor at T level below 15 nmol/L, 2) obesity below 12 nmol/L, 3) depressive moods, sleep disturbance, lack of concentration, and T2DM below 10 nmol/L, 4) erectile dysfunction and hot flushes below 8 nmol/L) (84–86), despite the well-known association between hypogonadism and bone impairment in men, the T threshold below which skeletal consequences begin is unknown (72). Some studies have suggested a compromised skeletal health and a more favorable response to T replacement for serum T levels below 6.94 nmol/L (52, 53, 87–89). Similarly, Finkelstein et al., utilizing different pharmacologic interventions to determine the levels of T and E2 which initiate the risk of bone loss in hypogonadal men, have shown that the relationship between gonadal steroid levels and bone impairment is likely represented by a continuum rather than a specific threshold. However, bone loss appears higher when serum E2 levels fall below 10 pg/ml and/or serum T levels fall below 6.94 nmol/L (72). A few years later, the same authors demonstrated that suppressing endogenous gonadal steroid production followed by a concomitant addition of escalating doses of T in 177 men aged 60 to 80 years old, resulted in a strong dose-response relationship between T and bone outcome measures (i.e., BMD and markers of bone resorption), but the adverse changes related to gonadal steroid deficiency begin at considerably varying levels of T concentration, although most outcome measures remain stable until serum T levels are well below the stated normal ranges (90).

The European Male Aging Study (EMAS) aimed at identifying clinical and biochemical criteria for diagnosing late-onset hypogonadism, concluded that this condition can be defined by the presence of at least three sexual symptoms (decreased sexual interest and morning erections and erectile dysfunction) in combination with a total T level of less than 11 nmol/liter (mainly below 8 nmol/liter) and a free T level of less than 220 pmol/L (91, 92). The occurrence of multiple end-organ deficits associated with androgen deficiency, such as lower hemoglobin, BMD, and lean body mass as well as reduced physical quality of life (suggesting a relationship between these symptoms and the degree of T deficiency), is especially more pronounced in men with severe than moderate forms of late-onset hypogonadism. Moreover, these features were less noticeable in men with low T only (without sexual complaints), highlighting the importance of sexual symptoms in defining late-onset hypogonadism (92).

The importance of the clinical, not only biochemical, criteria in defining hypogonadism, has identified the role of features like obesity and weight gain, together with grater age and lower education, as significant predictors for developing secondary hypogonadism, a condition which is potentially reversible in a substantial proportion of men by correcting these disorders. Conversely, a significant improvement in symptoms was not seen after the biochemical reversal of secondary hypogonadism to eugonadism (93). Moreover, while obese men with low total T (and low SHBG) but normal free T do not develop symptoms of hypogonadism and often remits to eugonadism, obese men with incident biochemical secondary hypogonadism, symptomatic androgen deficiency takes place when there is a combination of both low total and free T, suggesting to shift the focus not only on total T but also on free T in the diagnosis of hypogonadism, in order to avoid the over diagnosis of hypogonadism in obese patients (94).

## Type 2 Diabetes Mellitus and Bone

T2DM is associated with an increased risk of fractures in the spine and nonvertebral sites such wrist, foot, and even the hip (95). The fracture risk in T2DM appears to be higher with longer duration of the disease or poor glycemic control (96, 97). However, this increased fracture risk in patients with T2DM is paradoxically associated with normal or high normal BMD (18–20, 98–100). This implies that for a given BMD, subjects with T2DM have reduced skeletal strength, and are more likely to experience a fracture than age-matched individuals without T2DM. The pathogenesis of bone fragility in T2DM however has not been well elucidated yet (101).

There are several theories on why diabetes is detrimental to the skeleton, such as poor glycemic control leading to accumulation of AGEs, which increase the production of nonenzymatic cross-links within collagen fibers, therefore negatively influencing bone matrix properties and affecting bone remodeling, and directly activating inflammation and oxidative stress pathways that promote osteoporosis (23, 102). Moreover, changes in body composition, impaired bone healing, accumulation of microcracks and cortical porosity due to the loss of transverse trabecular connections (lower buckling ratios), the effect of the increased cortisol secretion, peripheral activation, and sensitivity (i.e., “cortisol milieu”) on the potential impairment of osteoblast activity, may all lead to lower bone turnover and poor bone microarchitecture (23, 95, 97, 103–107).

Other co-morbidities that may contribute to increased fracture risk in patients with diabetes include increased fall risk due to microvascular complications (i.e., neuropathy, vision loss, and balance impairment) from prolonged poor glycemic control, sarcopenia, and side effects from anti-diabetic drugs (23, 95, 108). In particular, the prolonged use of thiazolidinediones (TZDs), such as pioglitazone and rosiglitazone, which activate peroxisome proliferator-activated receptor gamma (PPARγ), has been associated with negative effects on bone metabolism, despite a beneficial effect on glycemic control (reduced insulin resistance and improved insulin sensitivity). Specifically, by activating PPARγ, the use of TZDs leads to increased adipogenesis, decreased osteoblastogenesis while at the same time increasing osteoclastogenesis, and promotes osteocyte apoptosis, therefore resulting in decline in bone formation with enhancement of bone resorption (23, 104, 109–112), consequently poor bone biomechanical properties.

Obesity, which often accompanies T2DM, represent another factor responsible of the increased risk of fracture. Although some studies have shown a positive correlation between total adiposity and BMD, justified by the increased loading together with the effects of leptin and increased aromatase activity which support bone formation (113, 114), after a certain point, increased adiposity can result in low BMD and increased risk for vertebral fractures (115, 116). In addition, obesity as well T2DM, are associated with increased inflammatory cytokines, such as TNF- α and IL-6 which have been shown to lead to osteoblast dysfunction. Their association with central or visceral fat deposition (the opposite of a peripheral fat metabolic profile) is considered to have the worse effect on BMD (117, 118). However, the effect of obesity on skeletal impairment is still controversial. Recent studies demonstrated that the weight loss resulting from bariatric surgery is associated with a reduction in bone density, which is attributed to changes in the hormonal secretion such as a reduction in leptin, estrogen and insulin, and potential vitamin D deficiency which can negatively impact bone health (113).

In T2DM, it is difficult to determine a specific threshold for glucose or glycosylated hemoglobin (HbA1c) above which skeletal complications begin due to existence of the above mentioned co-morbidities (23, 95, 108).

In fact, although some studies have shown a positive association between HbA1c and fracture risk in diabetes mellitus (both T1DM and T2DM) (97, 119), Vavanikunnel et al. reported that in patients with T1DM poor glycemic control with HbA1c above 8% was associated with an increased risk of fracture compared to those with HbA1c ≤ 7%. On the other hand, among patients with T2DM, the risk of fractures appears not be affected by HbA1c levels, supporting the notion that the risk of fracture in these patients might be related to risk factors that are independent of glycemic control (120). One possible explanation for this lack of association between poor glycemic control and fracture risk among patients with T2DM is the occurrence of insulin resistance and hyperinsulinemia in the early phase of the disease. In fact, since insulin is able to stimulate osteoblastogenesis, the effect of higher levels of insulin in the early stages of the disease might retard the influence of poor glycemic control on fracture risk in T2DM (21, 120). However the origin of these conflicting results can also be in part explained by the methodology used. In the study by Vavanikunnel et.al., long-term glycemic control was assessed as the a mean of eleven HbA1c measurements per subject with T2DM rather than from a single HbA1c measurement (120). In addition, other studies have also analyzed the risk of fracture using different approaches. For instance, the Rotterdam study assessed not only the incidence of fractures but also bone parameters, such as femoral neck and lumbar spine BMD, and hip bone geometry, demonstrating that the increased fracture risk in T2DM is driven by poor glycemic control and is associated with higher BMD and thicker femoral cortices in narrower bones (97). On the other hand, Vavanikunnel et al. assessed the risk of fractures by evaluating patients with a low-trauma fracture after diabetes onset, finding no involvement of poor glycemic control in increasing the risk of fractures in patients with T2DM (120).

To verify the existence of an association between poor glycemic control in T2DM and fracture risk, specifically a threshold of glucose or HbA1c at which bone impairment occurs requires a long-term follow-up of patients with early disease using periodic comprehensive measurements of bone health and metabolic parameters. This approach will allow a determination of the precise HbA1c level at which derangement in bone parameters occur and what happens long-term to bone parameters among those who stayed under good control *versus* those who will go on to have poor control, and taking into consideration the use of medications in addition to other relevant clinical factors such as age, duration of T2DM, and the existence of diabetic complications. Furthermore, if the population is substantial enough, information on the incidence of fractures may also become available, and again, whether good control will prevent these events.

Considering the normal to high normal BMD in T2DM, the cause of the skeletal fragility in these patients is likely a problem of bone quality rather than bone quantity (121, 122). The use of high-resolution peripheral quantitative computed tomography (HRpQCT) in the study of bone structure in subjects with T2DM has produced conflicting results. While some studies assessing the bone microarchitecture *in vivo* using HR-pQCT, support the hypothesis that bone fragility in T2DM is associated with increased cortical porosity (71, 123, 124), others report preserved microarchitecture in patients with T2DM (121, 125). In a small cross-sectional study of 19 elderly women with T2DM, cortical porosity at the radius and tibia was higher compared to controls. In addition, compressive bone strength, estimated using finite element analysis (FEA), was significantly lower in the radius (71). Data from the MrOS study, including 190 older men with T2DM, reported lower total bone cross sectional area (CSA) in the radius and tibia in diabetics compared to nondiabetics and reduced bending strength in cortical regions. However, bone strength was similar to that of controls in trabecular regions, with the higher trabecular volumetric BMD (vBMD) compensating for a lower total bone area in T2DM (126). In another study, diabetic women with history of fractures were found to have significantly higher cortical porosity, and lower bone strength (stiffness, failure load, and cortical load fraction) compared to diabetic women without fracture, whereas these indices did not differ between nondiabetic women and diabetic women without a history of fracture (123). Higher cortical porosity, lower cortical vBMD and tissue mineral density at the radius have been observed on a population of African American women with T2DM compared to nondiabetic controls (124). Likewise, in another study, increased porosity was reported in the midcortical and periosteal, but not endosteal, regions of the cortex. On the other hand, no significant differences were reported in cortical porosity in diabetic women without fracture compared to healthy controls (127). However, a small cross-sectional study on 51 subjects with T2DM, cortical bone deficits were only observed in a subgroup with microvascular complications (128), and could partly explain the lack of consistent findings between studies.

Despite the conflicting results with HRpQCT-derived FEA to estimate bone strength, studies using bone microindentation, a direct measure of bone strength have shown a consistent reduction in bone material strength index (BMSi) in diabetic subjects. Farr et al. conducted a study on 60 postmenopausal women, 30 with T2DM (duration >10 years), found that BMSi, which was inversely related to HbA1C level, was significantly lower in the diabetic women both before and after adjustment for BMI (121). Similarly, significantly reduced BMSi was reported in 16 postmenopausal women with T2DM when compared to controls, with a significant inverse correlation with the duration of disease and skin auto fluorescence, used as a surrogate for AGEs (129). Lastly, another study also found significantly reduced BMSi in women with T2DM, before and after adjustment for multiple covariates including BMI (122).

The most prominent impairment in bone metabolism associated with T2DM is decreased bone formation, with a reduction in the mineralizing surface, mineral apposition rate, and osteoblasts surface, resulting in an overall low bone turnover state (2, 20, 26, 130, 131). That bone disease in diabetes is characterized by low bone turnover is well established in both human and animal studies (20, 132–134). In a meta-analysis on individuals with diabetes (T1DM or T2DM), analysis of bone turnover markers, showed significantly reduced levels of serum osteocalcin (OCN) and C-terminal telopeptide (CTX), although other markers, including bone-specific alkaline phosphatase (BAP), did not differ from those without diabetes (20). Similar findings were reported in a subsequent meta-analysis from the same group (131). On the other hand, increased serum sclerostin levels (an inhibitor of bone formation) have also been observed in T2DM (135–137).

If the fracture risk in T2DM cannot be ascribed to lower BMD, it might be related from poor bone quality, and given the low bone turnover, likely due to the inability to replace an aging bone with new bone and repair microcracks, which increase with aging. The alteration in bone remodeling possibly from impaired cross-talk between osteoblasts and osteoclasts, likely led to low bone turnover, although the exact mechanism is still unclear (138). In a study on a rat model of T2DM, altered bone formation due to suppressed osteoblastogenesis has been identified as a key mechanism for impaired bone regeneration (139). *In vitro* studies of human immortalized bone marrow mesenchymal showed that stem cells (SCP-1) differentiated in the presence of serum from T2DM patients exhibited reduced osteoblast function, showing increased proliferation but impaired maturation with reduced alkaline phosphatase (ALP) activity and matrix mineralization compared to SCP1 cells differentiated in the presence of sera from non-diabetic control (either non-obese or obese). Moreover, altered gene expression of matrix components and osteogenic transcription factors was observed after stimulation with serum from patients with T2DM compared to serum from non-diabetic controls. Taken together, these results may suggest the existence circulating factors in the blood of T2DM patients capable of affecting osteoblastic function (140).

## Hypogonadism and Type 2 Diabetes Mellitus on Bone

To date, only a limited number of studies have investigated the effect of the combination of both hypogonadism and T2DM on bone health. Dhindsa et al. evaluated the relationship between T concentrations and BMD in men with T2DM (26). They were able to demonstrate that free and total T concentrations were positively associated with bone mineral content (BMC) in the arm, while free T was positively correlated with BMD in arms and ribs but not at other sites, such as hip, spine, or total body BMD, suggesting that T may have variable effects on BMD at different skeletal sites. This could also mean that hypogonadism may result in a more pronounced or rapid bone loss at certain sites such as the arms and ribs compared to the legs, hips, or the spine among men with T2DM (26). This study reported no significant difference in areal BMD (aBMD) at the lumbar spine, femoral neck, and total hip between eugonadal and hypogonadal subjects suggesting that among patients with T2DM, T deficiency may not have an effect on the BMD at these sites (26).

In a cross-sectional analysis, on a population of 105 hypogonadal men between 40 and 75 years of age, Colleluori et al., reported no significant difference in adjusted aBMD on the spine, total hip, and femoral neck between those with and without T2DM (141). However, using peripheral quantitative computer tomography (pQCT), they found that adjusted vBMD in those with T2DM was significantly higher compared to those without T2DM with bone CSA, whereas endosteal and periosteal circumferences significantly lower in the group with T2DM. These results suggest that hypogonadal men with T2DM have relatively higher vBMD but smaller bone size compared to hypogonadal men without diabetes (141). In the same study, analysis of the biochemical markers of bone turnover showed that serum CTX and OCN were significantly lower in hypogonadal patients with T2DM compared to those without T2DM. In particular, CTX levels were lower in patients with T2DM relative to their non-diabetic counterparts, while OCN levels were much lower in the group who has both hypogonadism and T2DM. These findings suggest that the suppression of bone turnover markers associated with T2DM prevails over effect of hypogonadism on markers of bone turnover. However on further analysis, this difference in the bone turnover markers between the groups disappeared with adjustment for medication use. In fact, the group of patients with T2DM on insulin (n = 23) had significantly lower OCN and CTX compared to untreated patients and those on medication other than insulin combined (n = 26). Simple regression analysis showed significant negative correlation between OCN, CTX, and HbA1c in the whole population. A separate analysis according to the presence of T2DM showed that the negative correlation between bone markers and HbA1c was observed only between CTX and HbA1c, and only among those with T2DM (141).

## Insulin Resistance: A Shared Feature in Hypogonadism and Type 2 Diabetes Mellitus

Insulin resistance is a feature that is shared by both T2DM and hypogonadism. Whereas insulin resistance is a defining characteristic of T2DM, it has a bidirectional relationship with hypogonadism, where it is both a cause and a resulting feature of this disease (8, 27, 142). Age-associated decline in T levels have been linked with lower lean body mass and insulin resistance in older men (143). T concentration is inversely related to BMI, diabetes and insulin resistance (9, 39, 144, 145); the latter a potent risk factor for both micro- and macrovascular complications of T2DM (146). Several studies have shown that reduced total T level is associated with insulin resistance, leading to the development of T2DM (28, 39, 147–149). T and insulin resistance seem to influence each other by creating a vicious circle in which insulin resistance is associated with a decrease in T secretion by the Leydig cells, which in turn can lead to worse insulin resistance and metabolic complications which further reduce T production (145).

Androgen deprivation in men with prostate cancer leads to central obesity and a deterioration in insulin sensitivity resulting in increased risk for T2DM (150, 151). Animal studies showed that targeted deletion of the androgen receptor in male mice leads to increased blood glucose levels from insulin resistance (152). Epidemiological studies in healthy adult men showed that fasting and 2-h plasma insulin were higher in the men with lower T compared to those with normal T even after adjustment for both BMI and waist/hip ratio (153). Treatments or modalities that improve systemic insulin resistance such as therapy with rosiglitazone or lifestyle modification leading to weight loss, have been found to induce a modest increase in T concentrations in men with T2DM or obesity, but were unable to reliably normalize T concentrations (154–156). A cross-sectional study among adult males with diabetes (type 1 and type 2) displayed concordant findings of an inverse relationship between T levels and insulin resistance, showing that in men with T2DM, insulin resistance (as estimated by the HOMA-IR equation) was independently associated low total T levels even after adjusting for age, BMI, treatment regimens, and other potentially confounding variables (27). In a subgroup of subjects in this same study followed-up longitudinally, changes in T levels over time were independently correlated with changes in insulin sensitivity estimated from glucose disposal rate in patients with T2DM. In addition, individuals with low T levels were also more likely to have a BMI higher than 30 kg/m2, elevated triglycerides higher than 1.7 mmol/liter, reduced HDL cholesterol levels, and higher hs-CRP levels, all indicators of MetS, therefore of insulin resistance (27). These findings support the hypothesis that circulating T levels in men with T2DM may be influenced by insulin sensitivity, and vice versa (27). Among men with T2DM, those with HH are less insulin sensitive than those without HH (157) but T supplementation may improve their insulin sensitivity (158–162), however results have not been consistent in this area.

The mechanism by which T supplementation improves insulin sensitivity has several potential explanations including increases in lean mass, decreases in fat mass, through suppression of lipolysis, resulting in decreased circulating concentrations of free fatty acids which are known to induce oxidative stress that interfere with insulin signal transduction (157, 163, 164). T treatment has been observed to increase the expression of insulin receptor β, insulin receptor substrate-1 (IRS-1), Akt-2, and GLUT-4 in adipose, and suppress inflammatory mediators that interfere with insulin signaling such as CRP, IL-1β, TNF-α, and leptin (157). All of these are positive effects of T therapy on the metabolic profile, and in addition to the improvement in hypogonadal symptoms in men with T2DM and hypogonadism, further support T therapy in these patients (20). On the other hand, a few trials investigating the effects of T replacement therapy on insulin sensitivity and diabetes, presented conflicting findings about the role of T in modulating insulin sensitivity (160, 165). While some showed significant or slight improvement in the glucometabolic parameters with T therapy in hypogonadal men (2, 157, 166), others found no improvement in either insulin sensitivity or insulin resistance and HbA1c after T therapy with gel (167–169). Furthermore, a study of 39 men aged 50–70 years with low T levels and T2DM treated with metformin monotherapy, randomized to T gel (n = 20) or placebo (n = 19) for 24 weeks, showed that the beneficial effect of T therapy on body composition was not accompanied by improved insulin sensitivity (170). However, other studies support the hypothesis that insulin sensitization by T is not an immediate effect and may be mediated by changes in body composition. This is supported by a study using hyperinsulinemic clamp to evaluate insulin sensitivity in response to T replacement which showed no change in glucose uptake by muscles during clamps at an early time point of 3 week (157). Two meta-analysis of studies involving observational and randomized clinical trials investigating the effect of T supplementation on body weight, body composition and metabolic endpoints, have both shown an improvement in body weight and composition (with a significant reduction in fat and with an increase in lean mass) accompanied by a more favorable glycometabolic profile (reduction in fasting glucose and insulin resistance) after T treatment particularly in younger patients and in subjects with metabolic disturbances (171, 172). Nevertheless, acute withdrawal of T administration in men with HH resulted in reduced insulin sensitivity in the absence of apparent or detectable changes in body composition, BMI or leptin levels suggesting the possibility that sex steroids could probably modulate insulin sensitivity (173).

Thus, considering the potential involvement of T in regulating insulin sensitivity, a compensatory increase in the expression of androgen receptor in a setting of insulin resistance in HH and T2DM is expected. However, contrary to this hypothesis, researchers found significantly decreased expression in androgen receptor, estrogen receptor, and aromatase among men who have T2DM and HH compared to eugonadal men, indicating that the hypogonadal state in T2DM is associated with diminished responsiveness to T and E2. Nonetheless, there is a reversal of these deficits following T replacement (174).

## Insulin Resistance and The Bone-Pancreas-Gonads Loop

A two-way relationship between insulin and bone has been suggested by preclinical studies, showing a vicious cycle in which bone influences insulin sensitivity, and may be in turn affected by insulin resistance (175–179). Bone formation and bone resorption are regulated by osteoblasts and osteoclasts respectively, through insulin signaling mediated by insulin receptors on their surfaces (180, 181). Insulin acts as an anabolic agent in bone leading to improvement in bone formation (presumably *via* a proosteoblastic mechanism), resulting in increase in bone density and bone strength (181–183). Moreover, osteoblasts express GLUT4, an insulin dependent glucose transporter, and its deletion in osteoblasts from *in vitro* experiments results in impaired insulin stimulated glucose uptake, while *in vivo*, osteoblast specific GLUT4 knockout mice (ΔGlut4) displayed hyperinsulinemia, insulin resistance, and increased peripheral fat deposition (177). As shown by animal studies, insulin deficiency is associated with abnormalities in bone microarchitecture, in particular reduced mineralized surface area, osteoid surface, mineral apposition rate, osteoblast activity and number, while insulin replacement prevented impairment in bone integrity, and improved bone strength (181). Evidences from bone cell cultures suggest that the insulin receptor on bone cells may be important in modulating bone homeostasis by regulating osteoblasts proliferation and differentiation, collagen synthesis, and ALP production (181–183).

Studies in osteoblast-specific insulin receptor knockout mice (*Ob-IR-/-*) suggest the existence of a mutual relationship between bone and pancreas, and that insulin signaling in osteoblasts activates transcriptional events that promote osteoblast proliferation, survival, and differentiation, resulting in increases in OCN expression. The increase in OCN, in turn enhances β-cell proliferation, thus insulin production, and regulates fat accumulation and sensitivity, therefore contributing to whole body glucose homeostasis (184, 185). *Ob-IR-/-* mice displayed defective osteoblasts differentiation, reduced number of osteoblasts, decreased serum levels of bone resorption makers, and impaired postnatal trabecular bone acquisition leading to low circulating undercarboxylated OCN and reduced bone accumulation (185). In osteoblasts lacking insulin receptor the defect in differentiation was accompanied by decreased Runx2 expression, an osteoblast specific transcription factor, and its binding to the OCN promoter. The suppression of Runx2 is mediated by Twist2, its inhibitor, which is upregulated when the insulin receptor in osteoblasts is lacking. These data suggest that insulin signaling in osteoblasts is able to regulate osteoblasts differentiation, and induces OCN expression by attenuating the negative actions of the Twist2 transcription factor on Runx2 (176, 185, 186). The addition of insulin to osteoblasts cultures increases osteoblasts proliferation and differentiation (187). In *Ob-IR-/-* mice the number of osteoclasts remained unchanged compared to controls, but their activity was significantly reduced due to increased expression of OPG, a decoy receptor for RANKL (185). All these findings suggest the possible role of insulin signaling as an anabolic factor for bone formation; however, the details of this mechanism are still unclear.

Metabolically, the deletion of insulin receptor in osteoblasts as represented in the *Ob-IR-/-* model, results in marked increased in peripheral adiposity accompanied by glucose intolerance, hyperglycemia, hypoinsulinemia, and target tissue insulin resistance caused by reduced undercarboxylated OCN (184, 185). *Ob-IR-/-* mice had 40% greater fat mass and 8% lower lean mass compared to control mice. Moreover after glucose injection *Ob-IR-/-* mice displayed significantly higher plasma glucose levels and significantly reduced serum insulin levels than controls. Insulin tolerance tests and gene expression analysis showed that *Ob-IR-/-* mice had severe insulin resistance with significantly decreased pancreatic β-cell mass and insulin expression. The infusion of undercarboxylated OCN improves metabolic abnormalities in these mice (185).

## Osteocalcin and The Bone-Pancreas-Gonads Loop

Several studies in mice and humans have proposed a novel function of the bone, once considered an inert system with mere structural functions, and now regarded as an endocrine organ regulating energy metabolism and glucose homeostasis (176, 188). OCN, a hormone secreted by osteoblasts, has been suggested to mediate these functions (176). It has been shown that OCN, especially the undercarboxylated form, has a direct effect on insulin secretion and stimulates the response to insulin in adipocytes and myocytes, thus improving insulin sensitivity (189). Undercarboxylated OCN directly regulates glucose metabolism in skeletal muscle by enhancing insulin-stimulated glucose uptake, upregulating Akt signaling by activation of ERK kinase (MEK) in differentiated C2C12 myotubes, and increasing glycogen and fatty acid catabolism in muscle fibers (190, 191). OCN signaling in skeletal muscle induces the translocation of the glucose transporter, GLUT4, to the plasma membrane, stimulating glucose uptake in muscle (191). Furthermore OCN regulates IL-6 expression in muscle, which in turn favors glucose uptake and fatty acid oxidation in muscle and promote gluconeogenesis and glucose release in the liver (192). OCN also stimulates adiponectin secretion in adipose tissue, as well as enhances insulin sensitivity in muscle, liver, and white adipose tissue (176, 184, 185, 193). Moreover, OCN has an indirect role in stimulating insulin secretion through increasing the production of the gut-derived hormone glucagon-like peptide-1 (GLP-1), an incretin released by intestinal endocrine cells (194, 195).

A study using OCN knockout mice (*Ocn-/-)* mice (OCN loss-of- function model) and Esp knockout (Esp-/-) mice (OCN gain-of-function model) to explore the function of OCN in β-cell and adipocytes, reported that OCN secreted from bone might be involved in whole body glucose homeostasis (176). Mice lacking OCN exhibit hyperglycemia and impaired glucose tolerance from significantly reduced pancreatic β-cell proliferation and insulin secretion (176). OCN may regulate insulin sensitivity *via* adiponectin, and mice lacking OCN are insulin resistant from reduced adipocytes expression of adiponectin, an adipocyte-specific insulin-sensitizing hormone (176). Furthermore, *Ocn-/-* mice were obese, with abnormal accumulation of visceral fat, and increased serum triglyceride levels, suggesting the role of OCN in regulating energy metabolism  (176, 196). It has been shown that the expression levels of insulin and adiponectin were significantly increased when OCN expression vector-transfected COS cells were cocultured with islets or adipocytes (176). In addition, recombinant OCN injection improved glucose tolerance, insulin expression in β-cells, increased β-cell proliferation and insulin secretion, augmented adiponectin expression, decreased fat mass, and prevented high fat diet-induced obesity and diabetes in WT mice (176, 197). Intermittent administration of OCN through daily injections was followed by a significant improvement in glucose tolerance and insulin resistance in mice fed with either normal or high fat diet, and reversal of hepatic steatosis induced by high fat diet, suggesting its role in regulating fat metabolism also in the presence of obesogenic environment. Moreover, there was an increase in the number of mitochondria in skeletal muscle, and increased energy expenditure, and protection against diet-induced obesity in these mice (198). Animal model of gain in OCN activity as in *Esp-/-* mice was associated with a metabolic phenotype opposite to that observed in *Ocn-/-* mice; therefore characterized by hypoglycemia and low blood glucose level after glucose injection, increased insulin expression and secretion, increased insulin sensitivity, and adiponectin expression in adipose tissue. Furthermore,* Esp-/-* mice showed decreased fat mass and serum triglyceride level, a resistance to high fat diet-induced obesity, and diabetes, as well as resistance to streptozotocin-induced diabetes. These metabolic alterations were completely reversed after crossing *Esp-/-* mice with *Ocn+/-* mice in *Esp-/-*;*Ocn+/-* mice (176).

Insulin and OCN seem to influence each other in a positive feedback loop between bone and pancreatic β-cells, in which insulin signaling in osteoblasts enhances OCN production and bioavailability (184, 185). *In vitro* experiments showed that OCN gene expression during osteogenic differentiation was negatively affected by hyperglycemia and insulin resistance owing to a reduction in the activity of the human OCN gene promoter. Conversely, exposure to OCN increase glucose-induced insulin secretion in rat INS-1 β-cells, a cell line that secretes insulin in response to glucose (199). Mice fed a high-fat diet experienced improvement in whole-body glucose homoeostasis when insulin signaling in the osteoblasts is increased, while the opposite is true if signaling is reduced. Furthermore, high-fat diet resulted in insulin resistance in osteoblasts, leading to a decrease in OCN activity and a decrease in insulin sensitivity in white adipose tissue and skeletal muscle (178).

Results from *in vitro* and preclinical studies have suggested that undercarboxylated OCN enhanced human β-cell function (200). In humans OCN has shown an inverse correlation with plasma glucose level and fat mass in elderly non-diabetic subjects and a positive association with insulin sensitivity and adiponectin levels (201, 202). These findings were supported by results from cross-sectional and longitudinal studies showing an inverse correlation between total serum OCN levels and HOMA-IR, and the changes in fasting plasma glucose (203). Moreover, in patients with T2DM serum OCN was inversely associated with glucose and visceral fat mass and positively with serum adiponectin levels, and parameters of insulin secretion and sensitivity. During treatment of T2DM, changes in OCN were negatively correlated with changes in HbA1c (204–206). Data from a cross-sectional study showed that OCN was a negative and independent predictor of HbA1c in Korean men and women younger than 50 years, although no such association was found in individuals aged 50 years and older (207). All of these findings were in agreement with results from a meta-analysis of observational studies, which reported a positive association between serum total OCN levels and β-cell function, while a negative correlation with HbA1c (208).

Since OCN is an osteoblast-specific secreted hormone and osteoblasts differentiation is regulated by T, this may be an important factor linking glucose homeostasis with gonadal steroids (65). It has been suggested that undercarboxylated OCN regulates fertility and possibly the production of sex steroid hormones, primarily in males (209, 210). While *Ocn–/–* female mice have been observed to have normal fertility, male mutant mice showed a decrease in testicular size, epididymis, reductions in the weight of reproductive organs, lower T levels, and oligospermia. Conversely, OCN administration improved these parameters of male fertility in these mice (78, 176, 210). Furthermore, the reproductive phenotype of the in *Esp−/−* male mice was opposite that of *Ocn–/–* (210). This difference in gender was also noted in humans showing a significant association between serum OCN and T levels during mid-puberty in males, but no association between E2 levels and OCN in females (211). This finding could also signify that OCN could be involved earlier during puberty and rapid skeletal growth in males. *In vitro* experiments also demonstrated that osteoblast-conditioned medium and undercarboxylated OCN increased T production by the testes but did not affect E2 or progesterone levels produced by ovarian explants (78). It was observed that in absence of OCN, Leydig cell maturation appeared to be halted suggesting that OCN might play a possible role in promoting T synthesis as shown in coculture assays, in which the supernatants of WT but not of *Ocn–/–* osteoblasts in culture increased T production by Leydig cells of the testes. In addition, OCN deficient male mice had low circulating T levels, compared high T concentrations in *Esp–/–* (210). Moreover, *Esp−/−* mice, which have increased insulin signaling in osteoblasts showed increased fertility, implying the possibility that male fertility may be regulated by insulin signaling in the osteoblasts (184). This finding has been supported by data from another study showing that insulin favors male fertility by stimulating bone turnover and osteoclasts-mediated activation of OCN (212).

OCN acts through a testis-specific putative receptor, identified as GPRC6A, which has been found to be expressed in different organs such as bone, pancreas, muscle, fat, testis, liver, and prostate (210, 213, 214). Some studies proposed a role for the loop between OCN, GPRC6A, and T in regulating energy homeostasis and male fertility (210, 212, 215–217). In fact, GPRC6A is involved in T biosynthesis in Leydig cells and in T-mediated stimulation of insulin secretion in the pancreatic islets (215), while β-cell proliferation and insulin production are regulated by pancreatic GPRC6A (216, 218). These observations suggests that insulin signaling in osteoblasts may regulate T biosynthesis in the testis though OCN and vice-versa. Studies in humans report an association between serum OCN and T levels supporting the reproductive role of OCN (211, 219). Additionally, in patients with primary testicular failure from a heterozygous missense variant in the transmembrane domain of GPRC6A (F464Y) resulting in interference in the GPRC6A signaling pathway, glucose intolerance accompanies the reproductive hormone deficit, a phenotype analogous to those found in ONC- and GPRC6A-deficient mice (176, 210, 212, 220). These results not only suggest that the pathway between OCN and GPRC6A is conserved within mice and humans but also propose a probable key role of OCN in regulating T levels in patients with T2DM (176, 210, 212, 220, 221).

## Conclusion

The impact of T2DM on the hypothalamic-pituitary-gonadal axis is manifested by a type of hypogonadism that is characterized by reduced or normal gonadotropins resulting in the so-called HH. The high prevalence of low T in patients with T2DM has led the Endocrine Society to recommend screening patients with diabetes mellitus for hypogonadism (222). Regardless of type or etiology, primary or secondary (such as HH), hypogonadism is associated with an increased risk for fractures. Given that T2DM is also associated with a higher risk for fractures, the combination of both could have worse consequence on bone health than either one alone. Considering the well recognized mutual and bidirectional association between hypogonadism and T2DM (2, 8–10), it is very likely that a significant number of men could be at a greater risk for fractures. However, while hypogonadism is associated with low BMD and high bone turnover, mainly from increased bone resorption (15, 16, 22, 41, 46, 47), T2DM is characterized by an opposite scenario including low bone turnover, more specifically reduced bone formation, with normal or high normal BMD (17–21). Despite this difference in bone phenotype, it is possible that both T2DM and hypogonadism will converge to promote an even greater increased risk for fractures. The very few investigators who examined the effect of the combination of hypogonadism and T2DM reported that men with both conditions have high or normal BMD but smaller bone size, with a significantly decreased bone turnover (141). Thus, the effect of T2DM on bone metabolism and BMD appears to prevail over the effect of hypogonadism, suggesting that impaired osteoblastogenesis and osteoblast function could be crucial determinants of bone quality in men who have a combination of both conditions. In fact, the effect of T in stimulating osteoblastic differentiation and proliferation, and inhibiting the apoptosis of osteoblasts and osteocytes suggest that when there is insufficiency of T, part of the mechanism leading to bone loss is impaired bone formation, unable to compensate for the increased resorption brought about by the reduction in E2 owing to reduction in substrate, i.e., T (48).

On the other hand, an emerging mechanism that may underlie the possible detrimental effect of the combination of T2DM and hypogonadism on bone formation could involve the osteoblast-specific hormone, OCN. In fact it has been hypothesized that the link between T2DM, hypogonadism and bone metabolism is the existence of a bone-pancreas endocrine feedforward loop. Insulin signaling in osteoblasts activates transcriptional events that promote osteoblasts proliferation, survival, and differentiation, which result in increased OCN production (223). Furthermore, OCN (in particular undercarboxylated OCN), not only has a positive effect on glucose metabolism by promoting insulin production and sensitivity, but also proposed to have a putative role in regulating fertility and the production of sex steroids, hence, an important bone-derived factor linking glucose homeostasis with gonadal steroids (223). Through its effect in regulating osteoblastogenesis, undercarboxylated OCN is in turn regulated by insulin. Hence, impaired insulin sensitivity, which has a key role in the development of T2DM, results in low OCN levels which lead to low T production in men which in turn is associated with insulin resistance completing the proposed bone-pancreas-gonads loop (8, 27).

**Figure 1:**
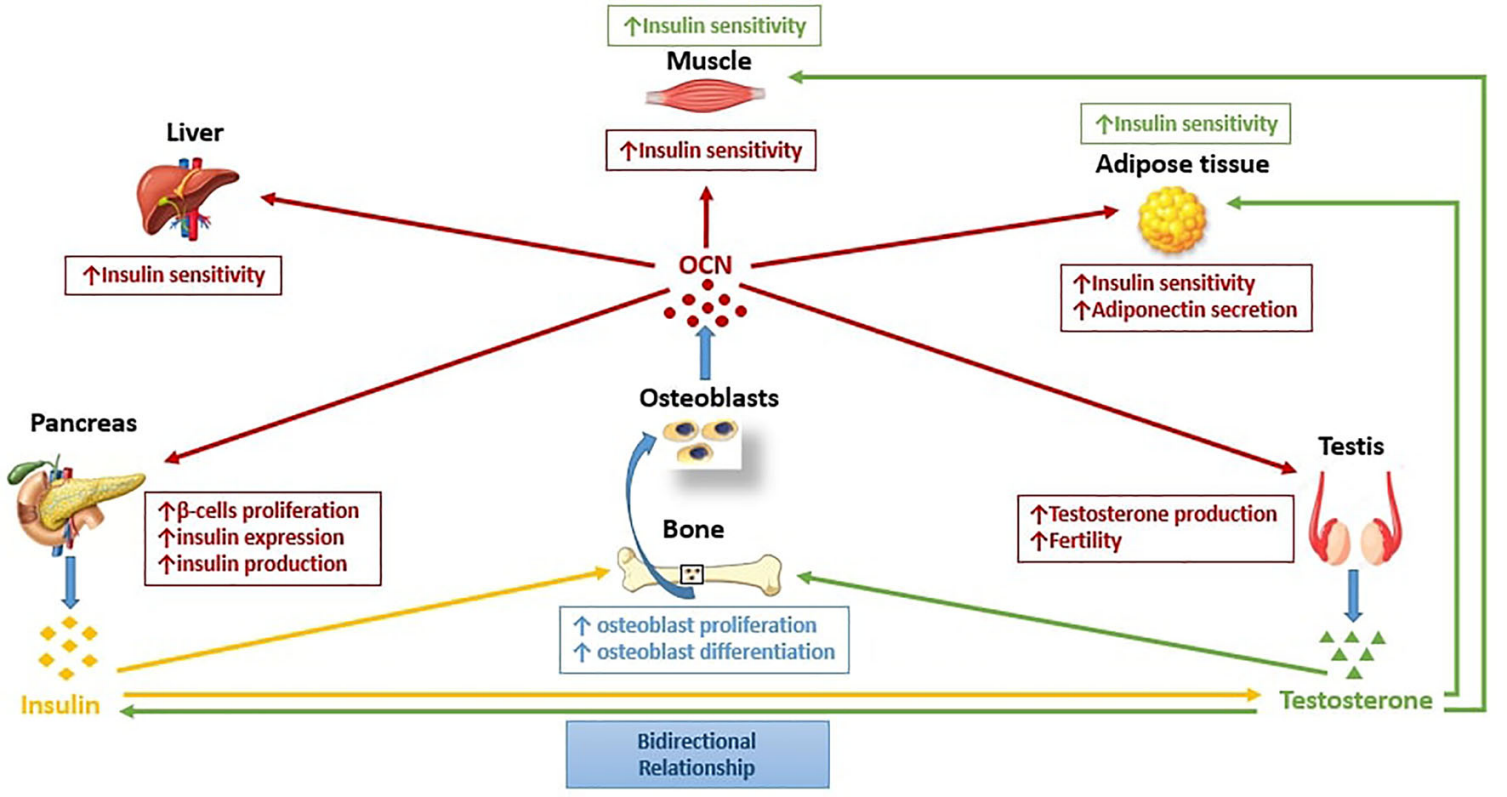
The pancreas-bone-gonads axis. Pancreas, bone, and testis communicates in a feedforward loop in which osteocalcin (OCN), a bone-derived hormone secreted by osteoblasts regulates glucose metabolism by stimulating pancreatic β-cell proliferation, insulin expression, and production, and improving insulin sensitivity in peripheral tissues, such as liver, muscle, and adipose tissue. Moreover, OCN regulates male reproductive function by increasing T production in Leydig cells. Insulin and T in turn, act on bone by enhancing osteoblasts proliferation and differentiation, and consequently favor OCN production from osteoblasts. Insulin and T are engaged in a bidirectional relationship in which T may improve insulin sensitivity, while circulating T levels may be influenced by insulin.

**Figure 1** summarizes our proposed framework for how the pancreas, gonads, and bone interact under normal physiologic state. Central to this interaction is the role of bone as an endocrine organ due to its role in generating osteoblasts which in turn produces OCN. OCN has multiple effects in several organs; on the pancreas by stimulating insulin production, on the testis by stimulating T production, and on the muscles, adipose, and liver by enhancing insulin sensitivity in these tissues/organs. In turn, the increase in insulin and T promotes increased osteoblastogenesis, and the cycle of further increase in insulin and T production, and enhanced insulin sensitivity from increased OCN is repeated. A disruption in any of the pathway of this proposed framework may lead to insulin resistance, which fosters the development of T2DM and hypogonadism. Although the metabolic complications from this combination is well-investigated, the skeletal complications remain poorly characterized. As stated in the previous paragraphs, preliminary data from the few studies that examined the effect of the co-existence of hypogonadism and T2DM on bone health, propose inactive bone turnover, and smaller bone size, suggesting poor bone quality. Whether improvement in insulin resistance or T supplementation restores bone turnover to normal, and improves bone quality remains unknown and deserves further studies. We however anticipate, (as shown in most secondary forms of osteoporosis), that an improvement in skeletal health will follow after treating the specific underlying cause.

## Author Contributions

VR: writing original draft, editing. RC: review–editing. RA-V: writing, editing. All authors contributed to the article and approved the submitted version.

## Funding

Merit Review 1 I01 CX001665 (RA-V).

## Disclaimer

The contents do not represent the views of the U.S. Department of Veterans Affairs or the United States Government.

## Conflict of Interest

The authors declare that the research was conducted in the absence of any commercial or financial relationships that could be construed as a potential conflict of interest.
